# The Influences of Drought and Land-Cover Conversion on Inter-Annual Variation of NPP in the Three-North Shelterbelt Program Zone of China Based on MODIS Data

**DOI:** 10.1371/journal.pone.0158173

**Published:** 2016-06-27

**Authors:** Dailiang Peng, Chaoyang Wu, Bing Zhang, Alfredo Huete, Xiaoyang Zhang, Rui Sun, Liping Lei, Wenjing Huang, Liangyun Liu, Xinjie Liu, Jun Li, Shezhou Luo, Bin Fang

**Affiliations:** 1 Key Laboratory of Digital Earth Science, Institute of Remote Sensing and Digital Earth, Chinese Academy of Sciences, Beijing, P. R. China; 2 State Key Laboratory of Remote Sensing Science, Institute of Remote Sensing and Digital Earth, Chinese Academy of Sciences, Beijing, P. R. China; 3 Plant Functional Biology and Climate Change Cluster (C3), University of Technology Sydney, Australia; 4 Geospatial Sciences Center of Excellence, South Dakota State University, Brookings, South Dakota, United States of America; 5 State Key Laboratory of Remote Sensing Science, Jointly Sponsored by Beijing Normal University and Institute of Remote sensing Applications of Chinese Academy of Sciences, Beijing, P. R. China; 6 College of geography, Chongqing Normal University, Chongqing, P. R. China; 7 Department of Earth and Environmental Engineering, Columbia University, New York, New York, United States of America; Chinese Academy of Forestry, CHINA

## Abstract

Terrestrial ecosystems greatly contribute to carbon (C) emission reduction targets through photosynthetic C uptake.Net primary production (NPP) represents the amount of atmospheric C fixed by plants and accumulated as biomass. The Three-North Shelterbelt Program (TNSP) zone accounts for more than 40% of China’s landmass. This zone has been the scene of several large-scale ecological restoration efforts since the late 1990s, and has witnessed significant changes in climate and human activities.Assessing the relative roles of different causal factors on NPP variability in TNSP zone is very important for establishing reasonable local policies to realize the emission reduction targets for central government. In this study, we examined the relative roles of drought and land cover conversion(LCC) on inter-annual changes of TNSP zone for 2001–2010. We applied integrated correlation and decomposition analyses to a Standardized Evapotranspiration Index (SPEI) and MODIS land cover dataset. Our results show that the 10-year average NPP within this region was about 420 Tg C. We found that about 60% of total annual NPP over the study area was significantly correlated with SPEI (*p*<0.05). The LCC-NPP relationship, which is especially evident for forests in the south-central area, indicates that ecological programs have a positive impact on C sequestration in the TNSP zone. Decomposition analysis generally indicated that the contributions of LCC, drought, and other Natural or Anthropogenic activities (ONA) to changes in NPP generally had a consistent distribution pattern for consecutive years. Drought and ONA contributed about 74% and 23% to the total changes in NPP, respectively, and the remaining 3% was attributed to LCC. Our results highlight the importance of rainfall supply on NPP variability in the TNSP zone.

## Introduction

The intensified anthropogenic emissions of carbon (C), particularly the burning of fossil fuels [1–3], is widely considered to be the main cause of recent global warming [1,2]. However, longer-term economic growth and social development cannot be achieved without adequate and affordable energy supplies, which will require continuing significant contributions from fossil fuels [4,5]. Previous studies have demonstrated that vegetation alone sequester about one third of C emissions from fossil fuel combustion [6–8], and net primary production (NPP) represents the amount of atmospheric C fixed by plants and accumulated as biomass [9,10].

Emission reduction targets have become an important issue at United Nations Climate Change conferences, and China is one of the major signatory countries for C emissions in the world [11]. China’s 2020 and 2030 emission targets are challenging, and scientists have extensively investigated potential ways to reduce China’s C emissions [12–14]. Terrestrial ecosystems represent a major sink in the global C cycle and help to slow the increase in atmospheric C levels [15, 16]. As terrestrial ecosystems offset C emissions, plants can contribute to emission reduction targets [2,17,18] and thus can be included in C tax calculations [19–21].

The Three-North Shelterbelt Program (TNSP) zone covers the northwest, north, and northeast regions of China, accounting for more than 40% of China’s landmass, and about half of this zone are barren or sparsely vegetated. Since 1978, the government has been carrying out an extensive afforestation exercises in the TNSP zone. These programs consist of three stages (1978–2000, 2001–2020, and 2021–2050) with the key objective being to increase regional forest coverage from 5% to 15% [22]. Several important ecological restoration programs were launched by the government in this region in the late 1990s and the early 2000s, and land cover conversion (LCC) was identified as one of the most significant results from those programs [23–27]. The TNSP zone consists of arid and semi-arid lands, and vegetation growth in this region is sensitive to drought conditions [28–31]. Several previous studies have focused on the effectiveness of these ecological restoration programs[23,27,32,33], but few have evaluated the role of LCC or drought on NPP.

Quantifying NPP variability and its causative factors have significant implications for the terrestrial C cycle [10, 34]. NPP variability relates to multiple mechanisms including climate factors and anthropogenic activities [9, 10, 35–39]. Assessing the relative roles of different causal factors contributing to NPP variability is of a great significance for establishing reasonable local policies to realize emission reduction targets for the government [40–43]. Therefore, our main objectives are (1) examine the temporal and spatial variations in NPP for 2001–2010, and (2) assess the relative contributions of drought and LCC to changes in NPP.

## Materials and Methods

### 2.1 Study Area

**Fig 1:**
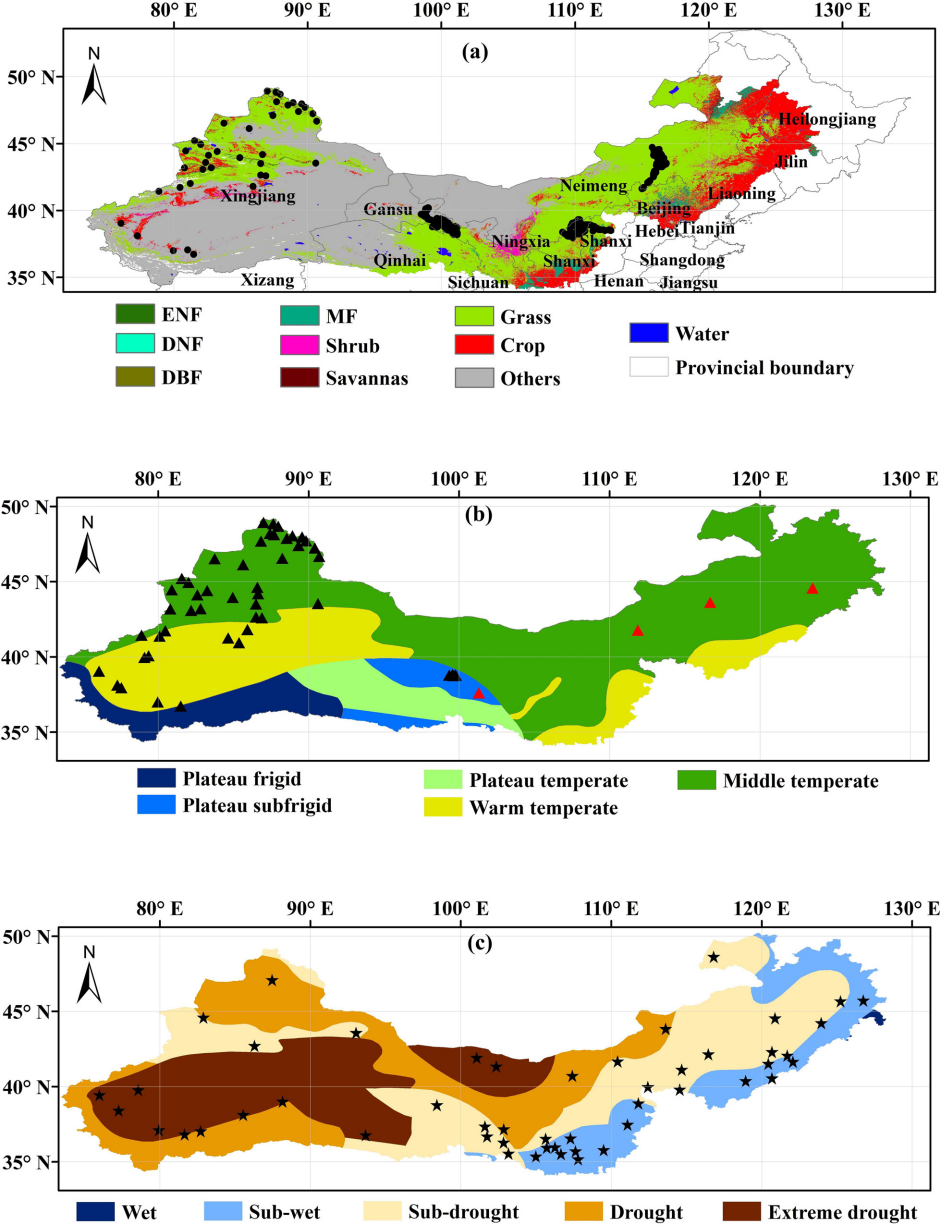
**Location of the study area, with (a) land cover distribution derived from MCD12Q1 in 2010, (b) temperature zone, and (c) drought zone**. The dots in (a) indicate the field observations for land cover validation. The black and red triangles in (b) indicate the field measured net primary production (NPP) and observations from 4 flux tower sites for MODIS NPP validation. The stars in (c) indicate weather stations where data are obtained to calculate Standardized Precipitation Index (SPI). Abbreviations in (a): Evergreen Needleleaf Forest (ENF), Evergreen Broadleaf Forest (EBF), Deciduous Needleleaf Forest (DNF), Deciduous Broadleaf Forest (DBF), Mixed Forest (MF). Closed Shrublands and Open Shrublands have been combined as ‘Shrub’; Woody Savannas and Savannas as ‘Savannas’; Croplands and Cropland/Natural Vegetation Mosaics as ‘Cropland’; and Permanent Wetlands, Urban and Built-up areas, Snow and Ice, and Barren or Sparsely Vegetated areas have been combined as ‘Others’.

The TNSP zone spans 551 counties in 13 provinces across northern China, covering an area of about 397×10^4^ km^2^. According to the MODIS land cover product (MCD12Q1) in 2010 (Fig 1A), grassland was the main land cover in this zone, accounting for 38% of the area. Crops accounted for about 11% and were mostly restricted to the northeast. Closed and open shrublands accounted for 1.8% of the area. Forest covered only 1.7%, with 80% of this consisting of mixed forest. The predominant land cover type was, in fact, ‘barren or sparsely vegetated’, which accounted for 45% of the total area (Fig 1A). This area represents a typical arid or semi-arid region, and has a mean annual rainfall below 450 mm [22].

### 2.2 Data Pre-Processing

#### 2.2.1 NPP product

MODIS annual NPP data over the study area for 2001–2010 were derived from MOD17A3 product, produced by the University of Montana’s Numerical Terra dynamic Simulation Group with a spatial resolution of 500m [10]. The accuracy of MODIS NPP data has been comprehensively validated in previous studies [44–50]. This particular MODIS NPP products is validated to “Stage-3”, indicating that the accuracy has been assessed and is appropriate for scientific applications [46, 50]. In addition, in this study, 51 field measured annual NPP and 6 annual NPP observations from daily gross primary production (GPP) measurements at 4 flux tower sites (Fig 1B) were further used to validate MODIS NPP. Since ground NPP is difficult to determine, for the 6 flux tower observation, we assume that NPP is ~47% of annual GPP based on the previous studies [51–54].

#### 2.2.2 Drought index product

For arid and semi-arid TNSP regions, precipitation (or drought) dynamics are the major climatic factors influencing NPP variability [22,28,31]. The Standardized Precipitation Evapotranspiration Index (SPEI) calculated from precipitation and temperature data based on normalisation of the Thornthwaite water balance model (Eqs 1–5) [55] was used in this study to assess impact of drought on NPP variability. SPEI has advantages over other widely used drought indexes such as the Standardized Precipitation Index (SPI) and self-calibrated Palmer Drought Severity Index (PDSI), because it has both multi-timescale and temperature variability capabilities [24, 56]. In this study, 500 annual precipitation data for 2001–2010 were obtained from 50 weather stations (Fig 1C), and were used to calculated SPI (Eqs 6–11) [57] to compare SPEI. Vicente-Serrano [58] found that vegetation activity during the germination period is mainly determined by the precipitation over the preceding last 12 months. A timescale of 12 months (or longer) is commonly used to monitor long-lasting dry episodes [59]. In this study, we used a monthly SPEI dataset for 2001–2010, covering a 12-month time scale (SPEI12), at a spatial resolution of 0.5° latitude/longitude. SPEI dataset were obtained from https://digital.csic.es/handle/10261/72264.

$$\text{SPEI}=\text{W -}\frac{{\text{C}}_{0}+{\text{C}}_{1}W+{\text{C}}_{2}W^{2}}{1+{\text{d}}_{1}\text{W}+{\text{d}}_{2}{\text{W}}^{2}+{\text{d}}_{3}{\text{W}}^{3}}$$ (1)

$$\text{W}=\sqrt{-2\ln(P)}\;\text{for}\;\text{P}\leq 0.5$$ (2)

$$\text{F}(\text{x})=[1+(\frac{\alpha }{\chi \text{-}\gamma }{)}^{\beta }{]}^{-1}$$ (3)

$${\text{D}}_{\text{i}}{=\text{R}}_{i}-PET_{i}$$ (4)

$$PET=16K{\left(\frac{10T}{I}\right)}^{m}$$ (5)

where *P* is the probability of exceeding a determined *D* value, *P* = 1 − F(x). If *P*> 0.5, then *P* is replaced by 1 − *P* and the sign of the resulting SPEI is reversed. The constants are *C*_*0*_ = 2.515517, *C*_*1*_ = 0.802853, *C*_*2*_ = 0.010328, *d*_*1*_ = 1.432788, *d*_*2*_ = 0.189269, and *d*_*3*_ = 0.001308. *α*, *β*, and *γ* are scale, shape, and origin parameters, respectively, for *D* values in the range (*γ* >*D* < *∞*). *R* is the monthly cumulative rainfall (mm). *T* is the monthly-mean temperature (°C); *I* is a heat index, which is calculated as the sum of 12 monthly index values *i*,

H(x)=q+(1-q)G(x) (6)

$$\text{G}(\text{x})=\frac{1}{\beta^{\alpha }\Gamma (\alpha )}\int_{0}^{\text{x}}x^{\alpha -1}e^{-x/\beta }dx$$ (7)

when 0< H(x) ≤ 0.5

$$\text{SPI}=\text{-}\;(\text{t -}\frac{c_{0}+{\text{c}}_{1}t+{\text{c}}_{2}t^{2}}{1+{\text{d}}_{1}\text{t}+{\text{d}}_{2}{\text{t}}^{2}+{\text{d}}_{3}{\text{t}}^{3}})$$ (8)

$$\text{t}=\sqrt{\ln\left\{\frac{1}{{\left[H(x)\right]}^{2}}\right\}}$$ (9)

when 0.5 < H(x) < 1

$$\text{SPI}=(\text{t -}\frac{C_{0}+C_{1}t+C_{2}t^{2}}{1+{\text{d}}_{1}\text{t}+{\text{d}}_{2}{\text{t}}^{2}+{\text{d}}_{3}{\text{t}}^{3}})$$ (10)

$$\text{t}=\sqrt{\ln\left\{\frac{1}{{\left[1.0-H(x)\right]}^{2}}\right\}}$$ (11)

H(x) or G(x) is the cumulative probability, Γ(*α*) is the gamma function. *α*, *β*, and *x* are shape, scale, and precipitation, respectively, *q* is the probability of a zero, *C*_*0*_, *C*_*1*_, C_*2*_, *d*_*1*_, *d*_*2*_, and *d*_*3*_ are defined the same values as Eq 1 [57].

Monthly 1km MODIS enhanced vegetation index (EVI) product (MOD13A3) for the period 2001 to 2010 was obtained from ftp://ladsweb.nascom.nasa.gov/. We bilinearly resampled the SPEI product and MODIS EVI to the resolution of the MODIS land cover data (500 m), and calculated the ratio of the monthly EVI to 12–month sum of EVI values. We took this ratio as the weight (Eq 12) of the drought influence on annual accumulated NPP, and created annual SPEI by monthly SPEI products (Eq 13).

$${\text{W}}_{\text{i}}=\frac{{\text{EVI}}_{\text{i}}}{\sum_{\text{i}=1}^{12}{\text{EVI}}_{i}}\times 100\%$$ (12)

$$\text{Annual SPEI}=\sum_{\text{i}=1}^{12}{\text{SPEI}}_{i}\times W_{i}$$ (13)

where *i* = 1, 2, … 12 indicates the month number.

#### 2.2.3 Enhancement of MODIS Land cover product

The most recent version of the 500m MODIS land cover product (MCD12Q1) for 2001–2010 was obtained from ftp://ladsweb.nascom.nasa. In this study, 45 field observations in Xinjiang, 1217 in Gansu, 85 in Shanxi, and 353 in Neimeng (Fig 1A) were used to validate MODIS land cover product. The investigated land cover types in Xinjiang and Neimeng are mainly forest and grassland, respectively. Crop, shrubland, and grassland are the major land cover types were investigated in Gansu and Shanxi. Information from the quality flag of MCD12Q1 was used to reduce the number of illogical transitions (see Table 1). To enhance the quality of the MCD12Q1 product, for pixels identified as having illogical transitions, the pixels were assigned to whichever land cover type corresponded to the higher classification accuracy. For instance, the International Geosphere-Biosphere Program (IGBP) land cover type in one pixel was 10 (Grassland) in 2001 and 2 (Evergreen Broadleaf Forest) in 2002 and the corresponding classification accuracies was 90% and 40%, respectively. The land cover transition that occurred between 2001 and 2002 in this pixel was considered illogical and we assigned IGBP type 10 to this pixel for 2002 based on the higher classification accuracy.

**Table 1:** International Geosphere-Biosphere Program (IGBP) land cover classification scheme (a) and illogical transition matrix (b). Illogical transitions in the matrix are labeled “X”. Adapted from ([60], the transitions from cropland (IGBP classes 12 and 14) and barren or sparsely vegetated land (IGBP 16) to forest (IGBP 1 and 3–5) have been taken as logical because of the known effects of several important Ecological Restoration Programs, including afforestation and the return of grain plots to forestry. A transition from IGBP 12, 14 and 16 to evergreen broadleaf forest (IGBP 2) is considered illogical because it is difficult for evergreen broadleaf forest to survive in the TNSP zone, which is characterized as an arid or semi-arid region.

(a)	(b)																	
IGBP land cover class	No	1	2	3	4	5	6	7	8	9	10	11	12	13	14	15	16	17
Evergreen Needleleaf Forest	1		X	X	X	X	X									X		
Evergreen Broadleaf Forest	2	X		X	X	X	X									X		
Deciduous Needleleaf Forest	3	X	X		X	X	X									X		
Deciduous Broadleaf Forest	4	X	X	X		X	X									X		
Mixed Forest	5	X	X	X	X		X									X		
Closed Shrublands	6	X	X	X	X	X										X		
Open Shrublands	7	X	X	X	X	X			X							X		
Woody Savannas	8	X	X	X	X	X	X									X		
Savannas	9	X	X	X	X	X	X		X							X		
Grassland	10	X	X	X	X	X	X		X							X		
Permanent wetlands	11	X	X	X	X	X	X									X		
Croplands	12		X				X	X	X							X		
Urban and built-up land	13	X	X	X	X	X	X	X	X	X	X	X	X		X	X	X	X
Cropland/natural vegetation mosaics	14		X													X		
Permanent snow and ice	15	X	X	X	X	X	X		X	X		X	X	X	X			
Barren or sparsely vegetated	16		X									X				X		
Water	17	X	X	X	X	X	X	X	X	X	X					X		

### 2.3 Correlation Analysis

The spatial patterns of 10-year averaged and the linear trend of MODIS NPP for 2001–2010 were used to examine the temporal and spatial variations in NPP in the TNSP zone, and their distributions over different land cover types. The relationship between annual NPP and SPEI for 2001–2010 was used to explore the response of NPP to drought dynamics. Land cover information derived from the MCD12Q1 product was categorized into 10 classes (Fig 1A). MODIS NPP and SPEI products were resampled to the same resolution of the land cover data using abilinear algorithm. The size of different land cover types for 2001–2010 was investigated, as well as the NPP in the corresponding regions, to analyze the impact of LCC on inter-annual variations of NPP. In addition, the livestock numbers for 2001–2010 in Inner Mongolia, Ningxia, Gansu, and Xinjiang, were collected from the Statistical Yearbook and were used to analyse the impact of grazing on variations of NPP.

### 2.4 Contributions of Drought and LCC to Changes in NPP

**Fig 2:**
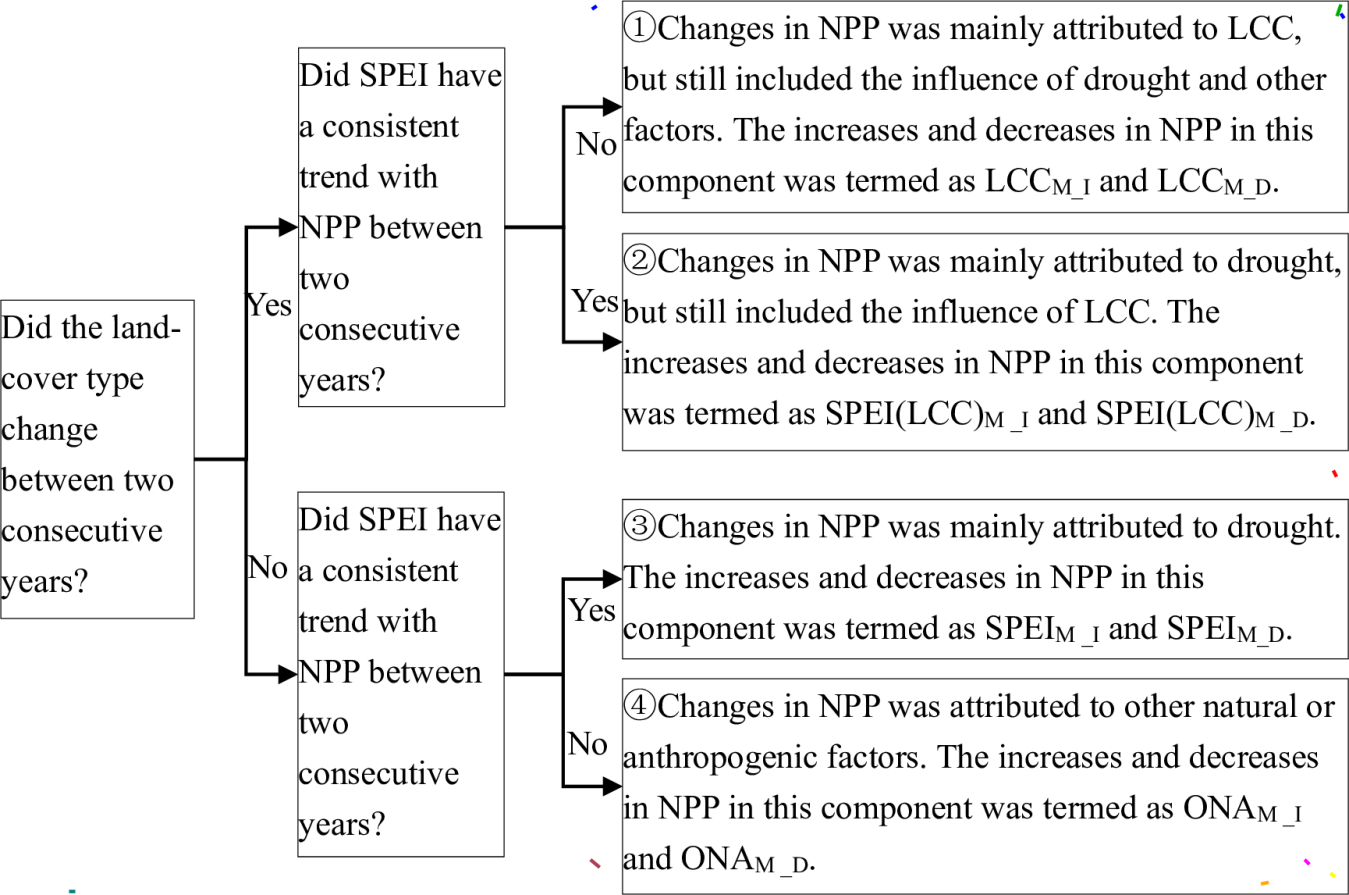
Flow chart for the decomposition analysis. The four components shown were used to find the relative contributions of drought, land cover conversion (LCC), and other natural or anthropogenic (ONA) factors to inter-annual changes in net primary production (NPP).

We introduced drought and LCC decomposition analysis (DLDA) method (Fig 2) to determine the relative contributions of drought, LCC, and other natural oranthropogenic factors (ONA) to NPP change between two consecutive years. In areas observed with LCC between two consecutive years and where NPP and SPEI showed a inconsistent trend, we attributed changes of NPP to LCC, and termed as LCC_M_ (①), which mostly excluded the drought influence from the role of LCC on NPP changes. In contrast, areas without LCC and with consistent NPP and SPEI trends, NPP changes were attributed to SPEI variations, and termed as SPEI_M_ (③), which mostly excluded the LCC influence from the role of the drought on NPP changes. SPEI(LCC)_M_ (②) represents areas with LCC and consistent NPP and SPEI trends, with changes in NPP attributed to interactions of LCC and SPEI variabilities. ONA_M_ (④) represented areas where NPP changes were attributed to other natural or anthropogenic factors, excluding drought and LCC, as LCC was not observed in these areas and inconsistent trends between NPP and SPEI in two consecutive years were found.

The increase and decrease in NPP due to these 4 components were calculated and then divided by the total NPP change, to derive the relative contribution rates (CR) of drought, LCC and ONA to NPP variability. Furthermore, the area percentage (AP) was used to quantify the area coverage of the influences of these components. We also calculated the total change in NPP between 2010 and 2001, as well as the AP (Eq 14) and CR (Eq 15) for each component.

$$AP_{\text{i}}=\frac{Area_{\text{i}}}{\text{Total}\;Area}\times 100\%$$ (14)

$${\text{CR}}_{\text{i}}=\frac{{\text{Changed NPP}}_{\text{i}}}{\text{Total changed NPP}}\times 100\%$$ (15)

where *i* represents a unique component in Fig 2; i.e. *LCC*_*M_I*_, *LCC*_*M_D*_, *SPEI(LCC)*_*M_I*_, *SPEI(LCC)*_*M_D*_, *SPEI*_*M_I*_, *SPEI*_*M_D*_, *ONA*_*M_I*_ or *ONA*_*M_D*_. The total change in NPP included increases and decreases in NPP series of consecutive years.

Finally, we compared the CRs of all components, and conducted a comprehensive analysis based on our findings and other studies to investigate which factors played the most dominant role in NPP variation for 2001–2010 in the TNSP zone.

## Results

### 3.1 Validations of MODIS NPP, MODIS Land Cover, and SPEI Products

**Fig 3:**
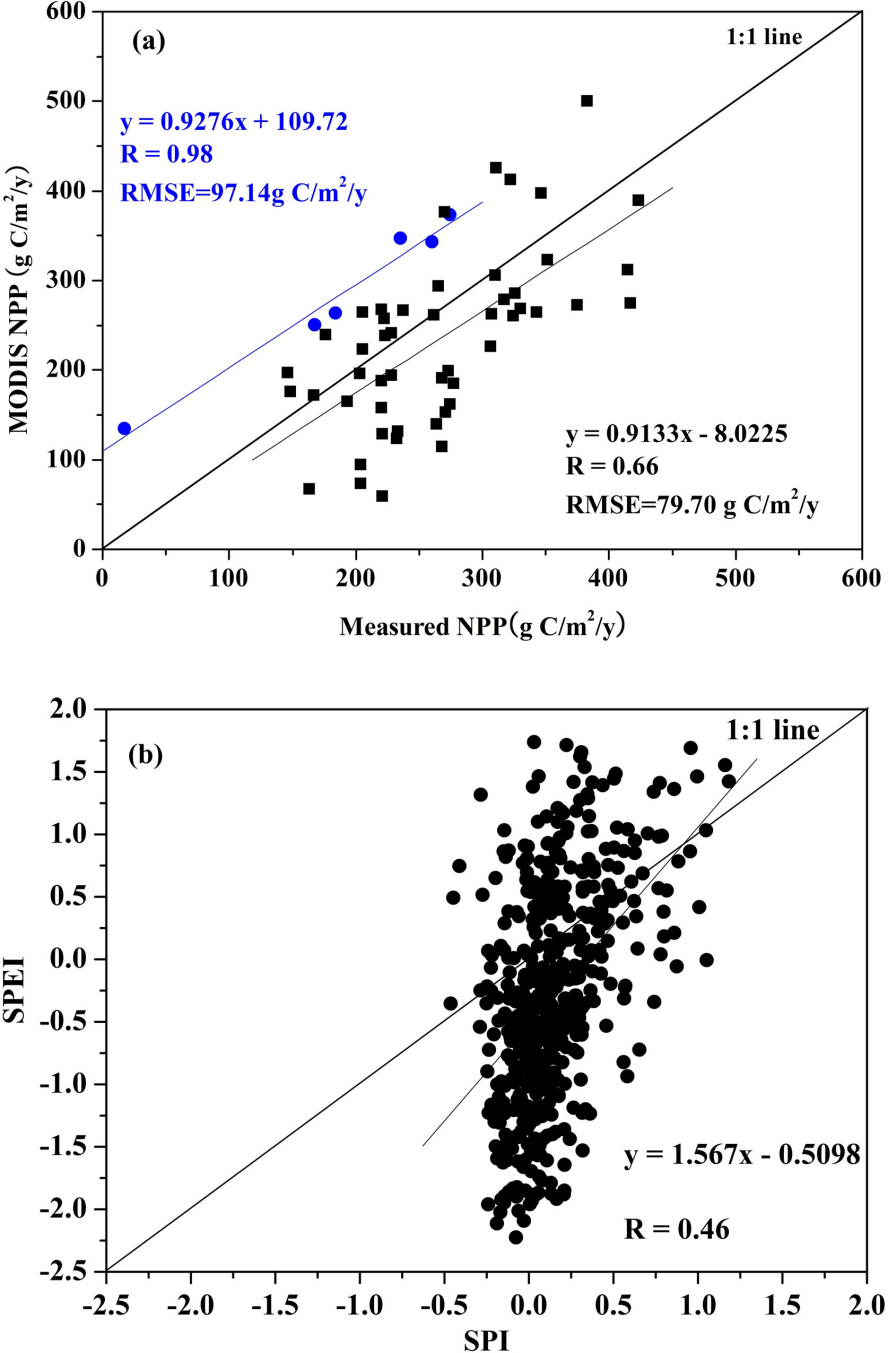
**(a) Comparison of MODIS net primary production (NPP) with reference flux tower observations and field measured NPP, and (b) the comparison between the Standardized Precipitation Evapotranspiration Index (SPEI) product and Standardized Precipitation Index (SPI) calculated from precipitation data over 50 weather stations**.

MODIS NPP is larger than the flux observations, and a significant correlation (p < 0.001) is found between them with a root mean square error (RMSE) of 79.70 gC/m^2^/y (Fig 3). MODIS NPP is also generally consistent with field measured NPP, largely following 1:1 line at annual levels with correlation coefficient (R) of 0.66 (*p* <0.001) and RMSE of 172.9 gC/m^2^/y for the overall dataset (Fig 3). Compared with the field observations, the accuracy of MODIS land cover is 69.5%, 76.8%, 71.3%, and 99.6% in Xinjiang, Gansu, Shanxi, and Neimeng, respectively. Although an obvious difference is observed between SPEI and SPI, they are still found to be generally consistent over 50 weather stations, with a R of 0.46 (p < 0.001).

### 3.2 Temporal and Spatial Variations of MODIS NPP

**Fig 4:**
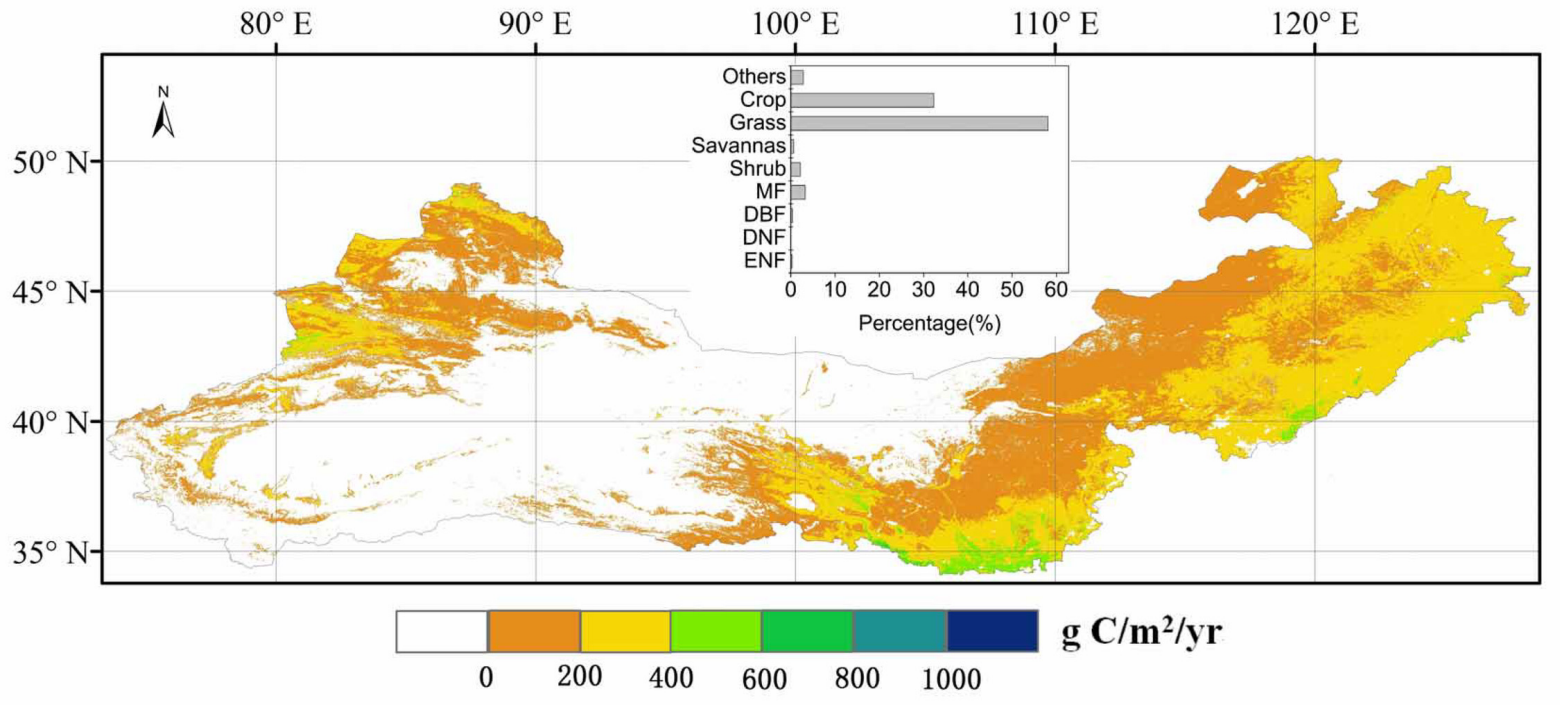
Spatial patterns of the 10-year (2001–2010) averaged MODIS net primary production (NPP). The bar graph shows the NPP distribution for different land cover types shown in the map.

**Fig 5:**
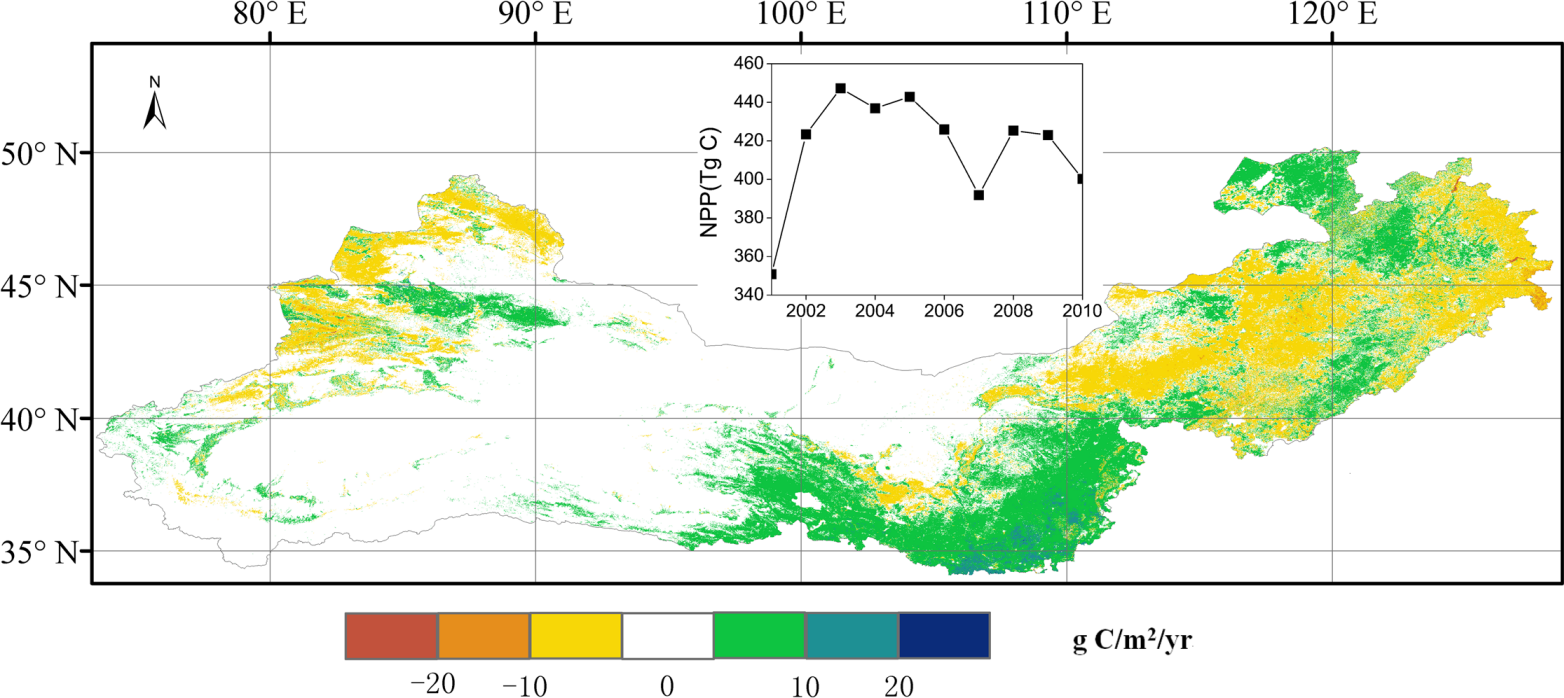
Spatial patterns of the temporal trend in terrestrial MODIS net primary production (NPP) for 2001–2010. The general annual values of NPP for 2001–2010 in the Three-North Shelterbelt Program (TNSP) zone are shown in the graph.

The mean NPP value in the TNSP zone for 2001–2010 was about 420 Tg C year^-1^, of which grassland and crops accounted for 58% and 32%, respectively, and mixed forest and shrub classes accounted for 3% and 2%, respectively. The large area of barren or sparsely vegetated land represented about 3% of the total NPP and the remaining land cover types contributed 2% of the total NPP (Fig 4). The average NPP value for grassland for 2001–2010 was about 160 g C m^-2^year^-1^, and the NPP value for the ‘crop’ class was around 300 g C m^-2^year^-1^ (Fig 4). Annual NPP values in sub-wet zone were generally larger than sub-drought and drought zones, and the annual NPP values in a large area charactered as drought or extreme drought were found to be close zero (Figs 1 and 4). In the south-central area, especially for the overlap area of sub-wet and warm temperate (Fig 1), the linear trend of MODIS NPP for 2001–2010 (Fig 5) showed that NPP increased by more than 10 g C m^-2^year^-1^. Grassland NPP values in the Inner Mongolia and Xinjiang regions decreased by up to 10 g C m^-2^year^-1^. The overall increase in grassland NPP was 1.5 Tg C year^-1^. Total mixed forest NPP increased by 0.5 Tg C year^-1^, largely due to an increase in the south-central area. There was a decrease in total crop NPP of 0.6 Tg C year^-1^, especially in northeast China. During the period 2001 to 2010, low NPP was observed in 2001, 2007, and 2010 (Fig 5).

### 3.3 The Correlations of NPP with Drought and LCC

**Fig 6:**
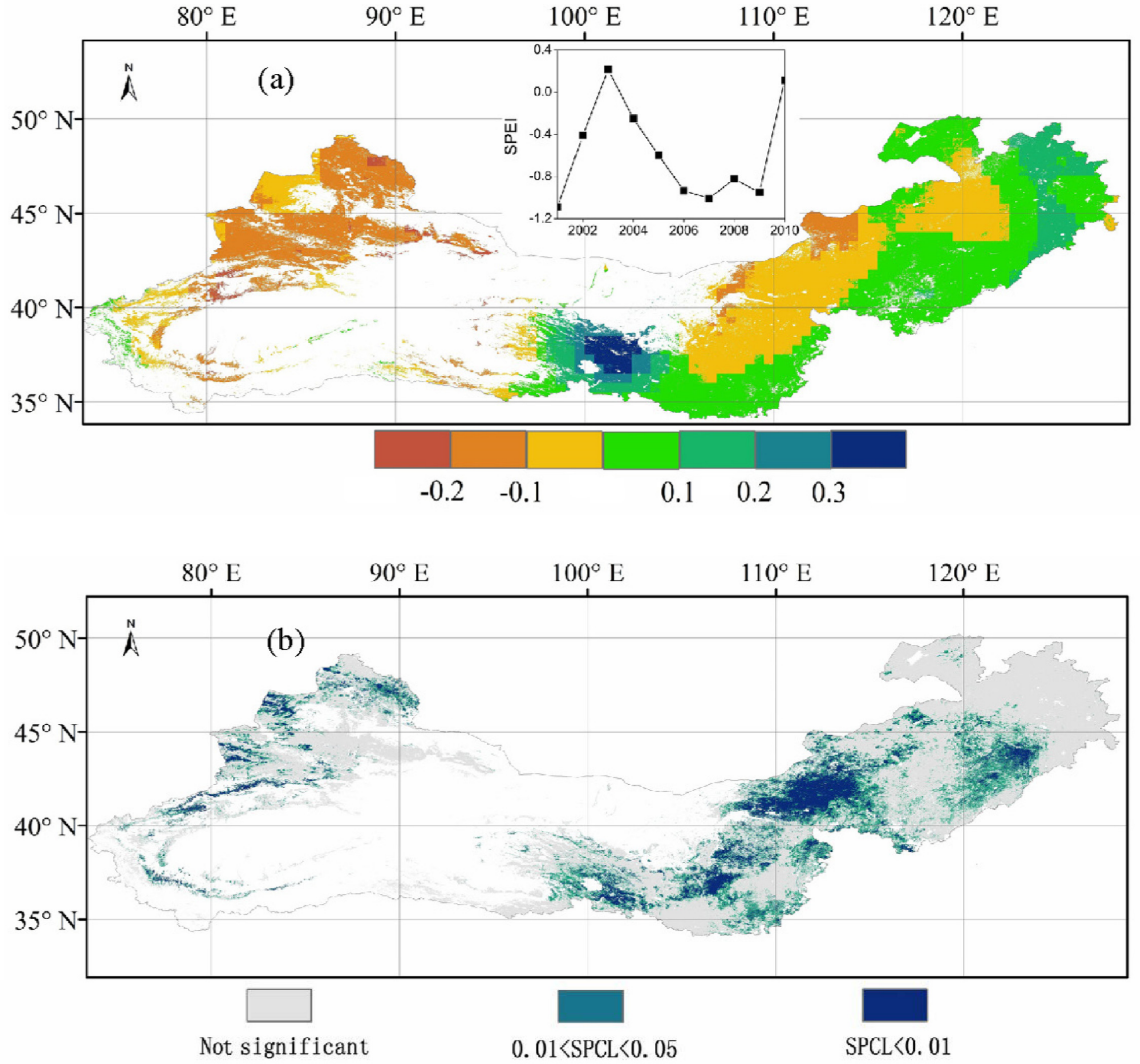
**The temporal trend of Standardized Precipitation Evapotranspiration Index (SPEI) for 2001–2010 (a), and the spatial patterns of the correlation between annual net primary production and SPEI for 2001–2010**. The general annual variation of SPEI for 2001–2010 in the Three-North Shelterbelt Program (TNSP) zone is showed by the line graph in part (a). SPCL in part (b) stands for ‘significant positive correlation level’.

There was a decrease in SPEI of about 0.1 year^-1^ for 2001–2010 for most areas in Xinjiang and Inner Mongolia (Fig 6A). For other areas in the TNSP zone, however, there was a general increasing trend of about 0.1 year^-1^, up to 0.4 year^-1^ in the area to the northeast of Qinghai Lake. The general annual variation in SPEI for 2001–2010 in the TNSP zone was found to be consistent with NPP variation (Fig 5), except for 2005, 2008, and 2010.The spatial patterns of the relationship between NPP and SPEI for 2001–2010 showed that about 60% of total annual NPP over the study area was significantly correlated with SPEI (P<0.05). For several grassland areas in Xinjiang and Qinghai, as well as large grassland areas of Inner Mongolia, there were a significant positive correlation (P<0.01) between NPP and SPEI (Fig 6B).

**Fig 7:**
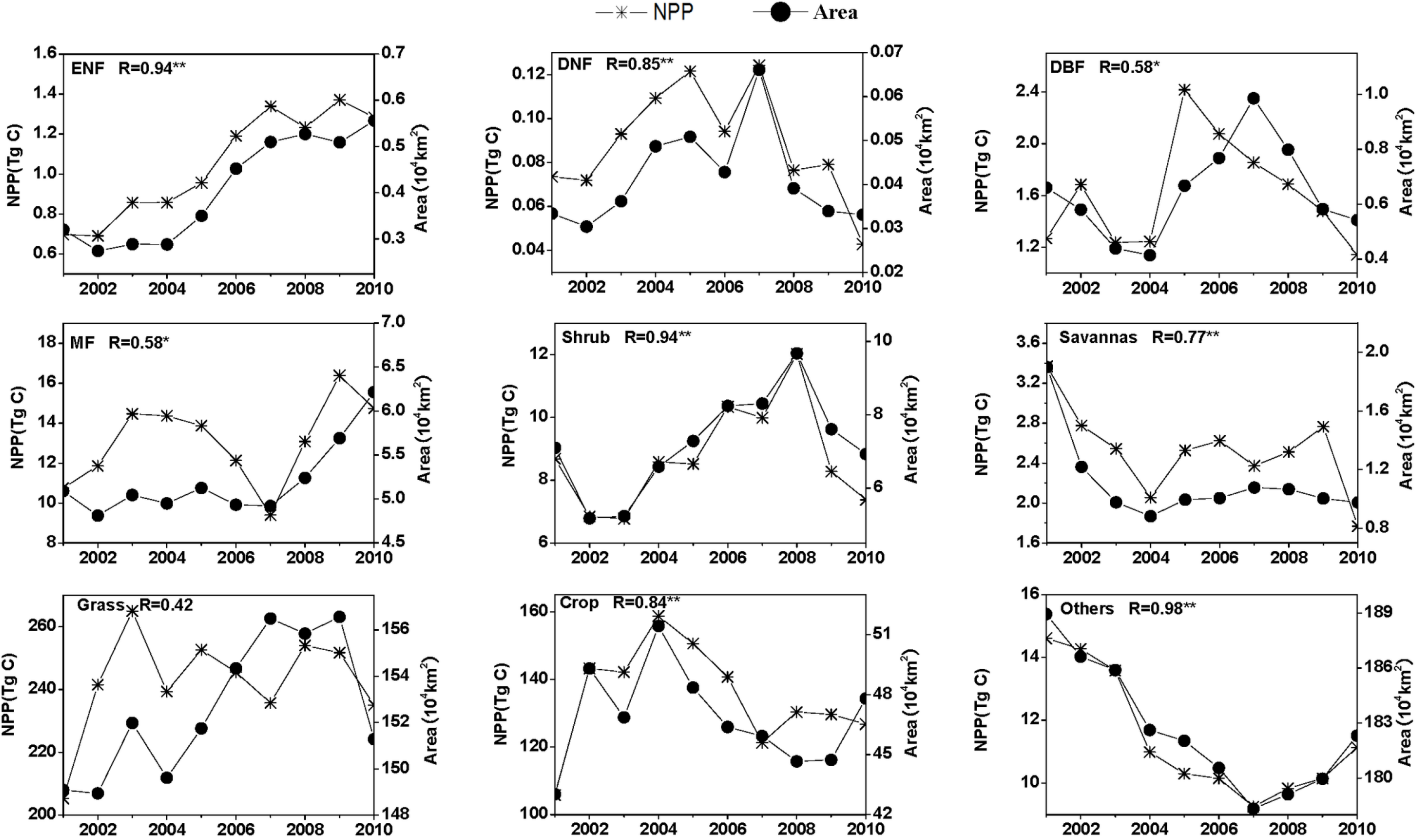
The relationship between total net primary production (NPP) values and land cover areas for 2001–2010. * and ** denote significant correlation at the 0.05 and 0.01 p-values, respectively.

The areas covered by different land cover types underwent significant changes in NPP during 2001–2010, especially in the case of barren or sparsely vegetated land-cover classes (Fig 7). The lowest NPP value for the ‘others’ class was found in 2007, when it was reduced by over 10×10^4^ km^2^ as compared to 2001, and the final reduction in area was 7×10^4^ km^2^ in 2010. The area of savannas decreased by 1×10^4^ km^2^. In contrast, the area of evergreen needleleaf forest increased continuously from 0.32×10^4^ km^2^ to 0.56×10^4^ km^2^, and that of mixed forest increased by 0.32×10^4^ km^2^. Grassland and crop area increased by 2.20×10^4^ km^2^ and 4.78×10^4^ km^2^ for 2001–2010, respectively (Fig 7). These results suggest that the Chinese ecological programs reduced the barren or desert areas, and led to land-cover conversions to more vegetated cover types. The relationships between total NPP and area for 2001–2010 for different land cover types showed a significant positive correlation (p<0.05), but not for grassland. Grassland NPP increased from 205 Tg C in 2001 to 235 Tg C in 2010. The largest loss (more than 5×10^4^ km^2^) of grassland area occurred between 2009 and 2010 (Fig 7). With a correlation coefficient of only 0.42, grassland area conversion showed no significant correlation with NPP variations at the 0.05 significance level.

### 3.4 The Relative Influence of Drought and LCC on NPP Inter-Annual Changes

**Fig 8:**
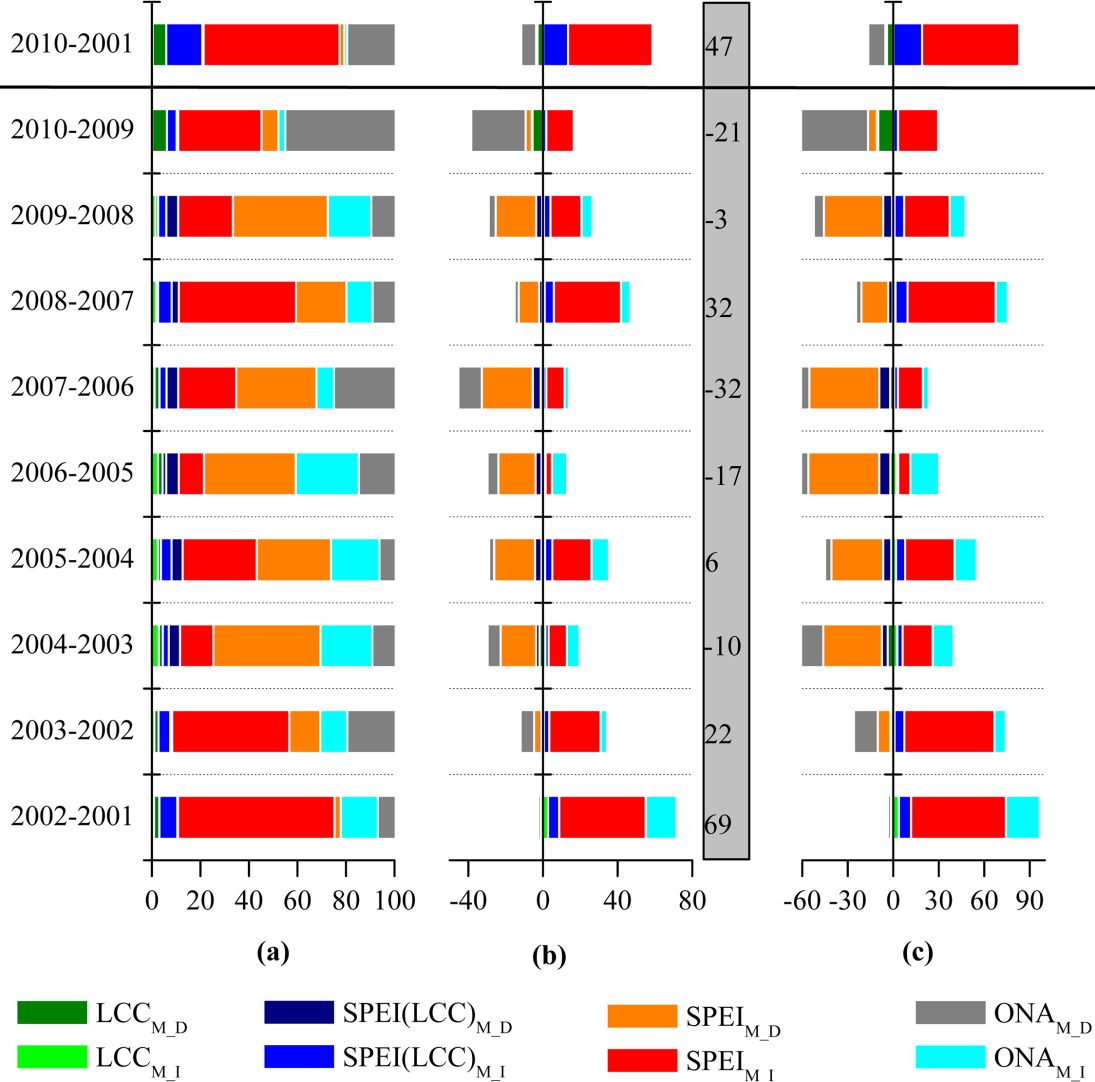
**The area percentage (AP) (a), change in NPP value (b), and contribution rate (CR) (c) for each component of the NPP variation for consecutive years**. The changes in NPP were mainly attributable to land cover conversion (LCC_M_), drought but still with some influences of LCC (SPEI(LCC)_M_), drought (SPEI_M_), or other natural or anthropogenic factors excluding LCC (ONA_M_). The subscripts *I* and *D* indicate increases and decreases, respectively. The values showing in the grey rectangular area are the net changes in NPP (Tg C) resulting from changes in NPP.

The results of the drought and LCC decomposition analysis are shown in Fig 8. Because several Chinese government ecological programs were being conducted in the TNSP zone, around 10% of the area was found to have undergone LCC between each pair of consecutive years. The largest and smallest amounts of LCC found were for 2004–2005 and 2002–2003, with the area percentage of 13% and 8%, respectively (Fig 8A). For most consecutive year pairs, NPP and SPEI-correlated changes occurred over 60% of the TNSP zone. Only two years (2005–2006, 55% and 2009–2010, 46%) were found with lower percentage. The area of NPP change caused by ONA was ~27% of the TNSP zone, and the percentage were 41% and 47% for 2005–2006 and 2009–2010, respectively (Fig 8A).

The lower values of the SPEI (Fig 6A), correspond to a greater contribution rate (CR) in reducing NPP, accounting for around 78% of the NPP decrease (Fig 8B and 8C). Conversely, higher values of SPEI for consecutive years, such as 2001–2002, 2002–2003, 2007–2008, and 2008–2009 (Fig 6A), correspond to a greater CR to increase NPP, accounting for 85% of the NPP increase (Fig 8B and 8C). The largest decrease and increase in NPP were observed for the consecutive year pairs of 2006–2007 and 2001–2002, the NPP values being 32 and 69 Tg C, respectively (Fig 8B). The CRs of our selected components in the TNSP zone to changes in NPP generally had a consistent distribution pattern for consecutive years; i.e., LCC, drought, and ONA contributed about 3%, 74%, and 23% of the total NPP change, respectively (Fig 8C). However, slight differences in the distribution patterns of the CR were still observed for some pairs of years. For example, for 2009–2010, ONA_M_ had the most significant contribution with a CR of 54% to the total NPP change (Fig 8B and 8C). In addition, from 2009 to 2010, the CR of LCC to NPP was the largest (10%). Meanwhile, SPEI_M_ and SPEI(LCC)_M_ had the smallest CRs, with values of 32% and 4%, respectively, to the NPP changes. According to the overall annual variation of SPEI for 2001–2010 in TNSP zone shown by the line graph in Fig 6A, the changes in SPEI and their CRs to NPP variations were highly consistent (Figs 6A, 8B and 8C). In the case of the changes in NPP between 2001 and 2010 in the TNSP zone, the CRs of LCC, drought, and ONA were 4%, 84%, and 12%, respectively. 21% of the area was found to have undergone LCC (Fig 8).

## Discussion

### 4.1 Possible Limitations of Products

According to the MODIS land validation strategy, stage 3 validation includes the spatial and temporal consistency assessments [61]. Stage 3 validation has been achieved for MODIS NPP product, for which uncertainty has been assessed, and are well quantified via independent measurements and other ancillary reference data. The global annual estimates of MODIS NPP is within 9.0% of average published results [62]. Our validation based on field measurements and observations from flux tower indicated that MODIS NPP was generally consistent with the reference data, and thus MODIS NPP product was suitable for analysis in this study [62].

A significant correlation (*p* < 0.001) was observed between SPEI and SPI, and an obvious difference was also found (Fig 3). SPI is calculated based on the amount of precipitation, and thus mainly indicate how much water is available for vegetation growth without the influence of temperature. However, SPEI not only accounts for the effect of temperature variability but it also accounts the lagged effects of previous months’ precipitations on the calculated drought [24, 56]. Additionally, annual MODIS NPP is calculated from gross primary productivity (GPP) and plant respiration. Thus, the value of annual MODIS NPP depends on the seasonal vegetation growth [45, 63]. Many studies have confirmed that the impacts of climate on NPP are strongly related to climate seasonal distribution and vegetation seasonal growth [30, 31,64]. Vegetation indices are widely used to assess vegetation growth conditions and plant productivity [65]. As the standard satellite vegetation product for Terra and Aqua MODIS data, the EVI provides improved sensitivity in high biomass regions while minimizing soil and atmosphere influences [66, 67]. In most of our study area, during the winter days, vegetation photosynthetic activities were inhibited because of cold temperatures and frozen soil, and NPP values were almost zero. Therefore, the influence of drought on annual accumulated NPP had different weights during the different stages of vegetation growth. In this study, EVI was used to assess the process of vegetation growth and calculate annual SPEI, and the drought had the most significant influence on annual NPP in the period with the maximal EVI values. Therefore, uncertainties due to SPEI in this study are considered marginal.

MODIS land cover type was produced via a supervised classification algorithm that was based on high-quality land cover training sites and was developed using high-resolution imagery in conjunction with ancillary data [68]. The primary land cover scheme was provided by an IGBP land cover classification [69–71]. We used the most recent version of the MODIS land cover type product, which included the adjustments for significant errors previously detected. The quality flag of MCD12Q1 showed that the accuracy of the IGBP land cover classification in the TNSP zone was around 80% for the period 2001 to 2010, and our validations by the field investigations showed that the grassland in Neimeng had a high accuracy, and a relative low accuracy was observed in forest (Xinjiang) or farming pastoral regions (Gansu and Shanxi). However, illogical land cover transitions were still observed for all consecutive pairs of years [60]. The definition of an illogical transition was adopted from previous study [60] and the illogical transition matrix (Table 1) was modified. The China State Forestry Administration has announced that afforestation and the return of grain plots to forestry took place over a large area of the TNSP zone [22, 33]. Thus, we took the transitions from cropland (IGBP classes 12 and 14) and barren or sparsely vegetated land (IGBP 16) to forest (IGBP 1 and 3–5) as being logical. However, a transition from IGBP 12, 14 and 16 to evergreen broadleaf forest (IGBP 2) was considered illogical, because it is difficult for evergreen broadleaf forest to survive in the TNSP zone, which is characterized as an arid and semi-arid region [9, 22, 30]. For each pair of consecutive years, we found that about 0.5% of the area of the TNSP zone was identified as illogical transitions.

### 4.2 Method of Assessing the Relative Influence of Drought and LCC on NPP Variability

Understanding the influences on NPP is one of the fundamental aims of global change research and is very important for governmental ecological policy making [41,43]. Previous studies have used regression modelling and scenario simulation methods to assess the relative role of different drivers on NPP variability [40, 41, 43, 72–75]. Regression models require fewer ecosystem-specific parameters, and generally provide the relationships of NPP with these parameters in order to explore the sensitivities of NPP to climatic or anthropogenic factors. However, these models are qualitative analysis, and are not able to determine the contributions of multiple mechanisms on NPP variability [40, 76]. Simulation methods are applicable under hypothetical steady-state conditions and rarely take into account the interactions between different drivers of NPP changes [34]. For example, NPP is estimated by assuming constant climate while changing other mechanisms to isolate the role of climate, but it overlooks the interactions among climate and other mechanisms [9]. Our method was based on both qualitative and quantitative analyses. In particular, we used the method of the decomposition analysis to separate the relative contribution of drought, LCC, and ONA. The relationships between NPP and SPEI and between NPP and LCC qualitatively described the responses of NPP to drought and LCC. We found that most NPP values were significantly correlated with SPEI (P<0.05), especially for grasslands, and that NPP generally showed a consistent change with LCC for all land cover types.

However, our method has some limitations as it does not completely separate the influences of drought and LCC on inter-annual changes in NPP due to the following currently insurmountable issues;

1. Lack of higher spatial resolution remote sensing and climate data. 500m resolution MCD12Q1 should be sufficient for grassland in the TNSP zone, but can hardly monitor the land cover change in the transition zone between crop and nomadic and forest areas. This transitional region is very large in the TNSP zone (Fig 1), and one cannot accurately extract the corresponding NPP using 500m resolution NPP data. Furthermore, the spatial resolution of climate data is also coarse, especially for the north-eastern and north-western mountain areas in the TNSP zone.
2. Lack of available spatial climate and anthropogenic data. Natural factors and anthropogenic activities, including grazing, cropping patterns, CO_2_ fertilization, vegetation growth, and nitrogen deposition are not available yet. We collected the livestock numbers in the four main provinces regions from Chinese statistical yearbook, and these data were collected at plot level, and could not be used to define the role of grazing on NPP changes spatially. Grazing affects NPP in theory, but the data at the province level dilutes this effect. Without other spatial climate and anthropogenic data, our decomposition analysis cannot qualitatively provide the CRs of more mechanisms to NPP variability.
3. LCC, SPEI and ONA might interactively influence NPP. In our proposed decomposition analysis method, four key components were produced, and separated the influence of drought and LCC on NPP changes. However, the drought still impacts annual NPP for components LCC_M_(①) and ONA_M_(④), although NPP and SPEI showed an inconsistent trend. In addition, ONA includes many climate and anthropogenicfactors, which might influence the changes in NPP for all components.

### 4.3 The Role of Drought and LCC in NPP Variability

As a climatic drought index, SPEI involves a climatic water balance [57]. In this study, SPEI was used to examine the role of drought on NPP variations. Mohamed et al. [77] found that temperature and precipitation significantly contributed to inter-annual NPP variability, especially for forests and grasslands. In this study, the highest NPP values were observed in the overlap regions of sub-wet and warm temperate zone (Figs 1B, 1C and 5), and a significant positive SPEI–NPP relationship was found for most grassland areas in the TNSP zone (Fig 6), indicating that climate plays a very prominent role on grassland NPP variation. We also observed that NPP spatial patterns were highly related to the drought, such as the highest and lowest NPP values were found in sub-wet and extreme drought areas, respectively (Figs 1C and 5), indicating that water availability play the major role on NPP in TNSP zone. Our results are consistent with many previous studies [39, 78, 79]. Bai et al. [30] and Guo et al. [31] found that the aboveground NPP was increased with increasing annual precipitation in Neimeng, and Peng et al. [80] found that precipitation was the major climatic factor influencing NPP variations in Xinjiang. In this study, NPP also showed significant correlations (p<0.01) with SPEI in large area in Neimeng and Xinjiang (*p*<0.01) (Fig 6). The increasing and decreasing SPEI caused the corresponding variations in NPP (Figs 6A and 8B). An increase in SPEI led to increased NPP. These results confirm that the TNSP zone is a water-constrained area, which agrees with the results of Nemani *et al*. [9]. Our results also showed that the CR of SPEI was higher than that of LCC and ONA, which also demonstrates that sufficient rainfall supply plays a major role in plant growth in arid and semi-arid regions and will increase NPP [10, 28, 29, 39, 42, 81].

LCC is the most obvious consequence of anthropogenic activities and constitutes a major factor affecting NPP [82–84]. During 2001–2010, LCC occurred in the TNSP zone due to anthropogenic activities including afforestation, deforestation, urbanization, restoration of farmland to forest and the return of grazing land to grassland [25–27, 31,33]. We found a increase in forest NPP and a decrease for the ‘others’ land-cover class, and strong LCC–NPP relationships for the evergreen needleleaf forest, deciduous needleleaf forest, shrub, savanna, crops, and others. This is in good agreement with previous studies which showed that human activities had a large influence on total NPP [85]. Several studies have assessed the effectiveness of Chinese ecological restoration programs within the TNSP zone [23, 27, 32, 33], but few have evaluated the effects of these programs on plant C sequestration. During the period 2001 to 2010, the area with LCC was around 10% of the whole TNSP zone. The changed NPP due to LCC was smaller compared the total changed NPP, and the CR of LCC to the NPP variation was about 3%. However, we found an increase in forest area for 2001–2010, resulting in a corresponding increase of NPP. The positive LCC–NPP relationship, which is especially evident for forests in the south-central area, indicates that ecological programs have a positive impact on C sequestration in the TNSP zone, which has also been confirmed by Yang et al. [85] about the influence of land cover change on NPP in Xinjiang. Additionally, the government policy to increase the forested area started in 1978, which is much earlier than 2001, when we started tracing the changes. Therefore, our study most likely underestimate the government’s effort to increase C sequestration since we started much later.

**Fig 9:**
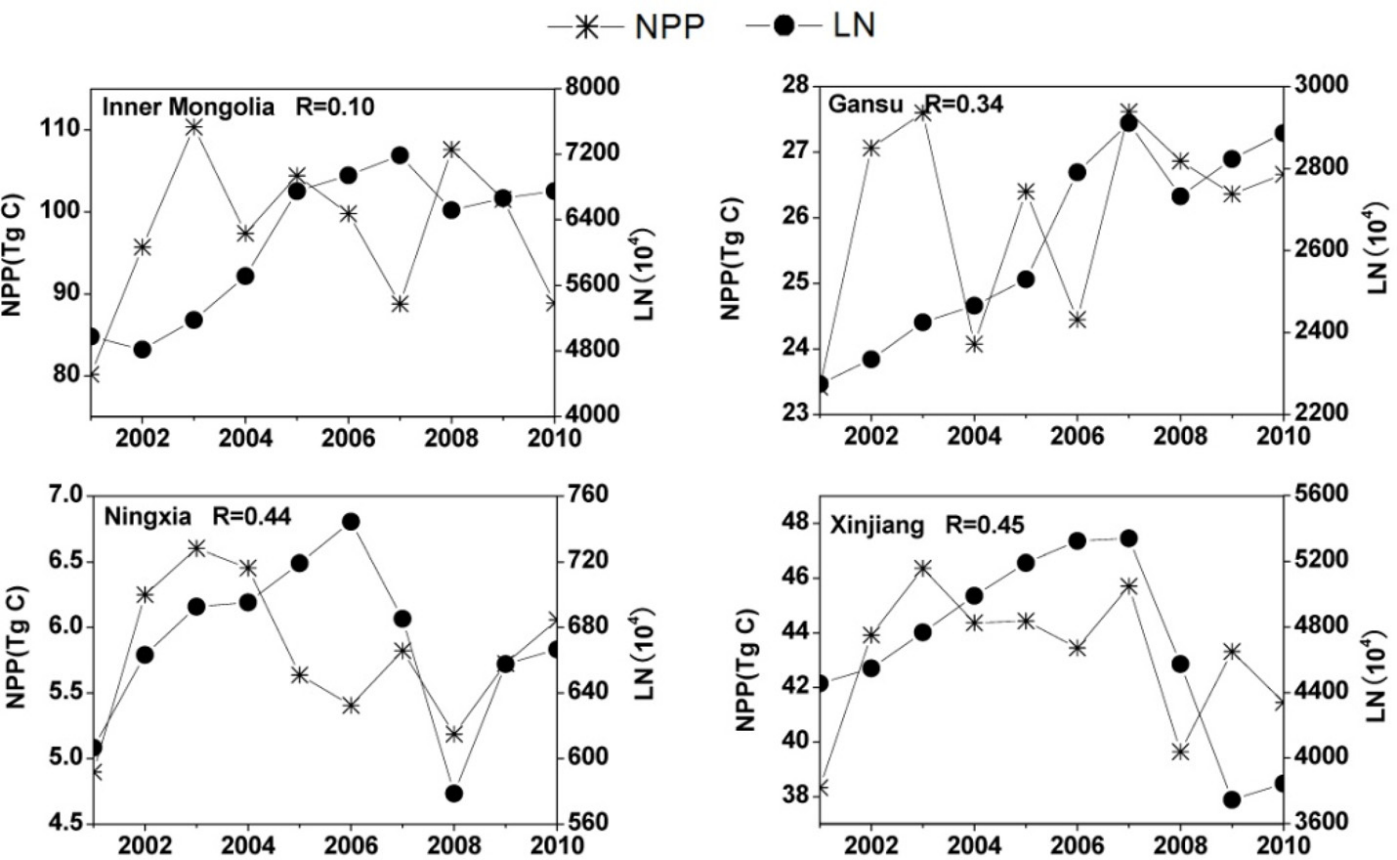
The relationships between total grasslandnet primary production (NPP) values and livestock numbers (LN) for 2001–2010, in Inner Mongolia, Ningxia, Gansu, and Xinjiang.

ONA contributed about 23% of the total change in NPP. Grassland is the main vegetation in the TNSP zone and grazing is an important and typical ONA factor. Grazing probably happened in areas where there was no LCC for 2001–2010. According to Fig 1, the study area almost completely covered the territories of Inner Mongolia, Ningxia, Gansu, and Xinjiang, where grassland NPP was more than 70% of the total grassland NPP in the TNSP zone. These four provinces were selected for analysis of the influence of livestock numbers on grassland NPP. The livestock numbers in these four provinces showed significant variations. This was especially the case in Inner Mongolia, where livestock numbers increased from 4800 ×10^4^ to 7200 ×10^4^ over the 10 years (Fig 9), as well as in Gansu province (increased from 2270 ×10^4^ to 2880 ×10^4^). Generally, higher livestock numbers lead to a decrease in total grassland NPP if other influences are ignored. However, the correlation coefficients for the relationships between total grassland NPP values and livestock numbers for 2001–2010 were only 0.10, 0.44, 0.34, and 0.45 in Inner Mongolia, Ningxia, Gansu, and Xinjiang, respectively. None of them were statistically significant (*p*<0.05), indicating that livestock numbers were not the major factor influencing on grassland NPP changes.

Previous studies confirmed that multiple mechanisms interactively influence NPP, and different regions have very distinct dominant drivers [9, 35, 36, 39, 85]. We found that water availability plays the main role on NPP variability in the TNSP zone, and LCC showed limited influence on NPP changes because of the small area observed with LCC compared the whole TNSP zone. These findings indicate that government policies should support to improve water management in those drought-prone zone. Irrigation or other methods of expanding water supply would be the major policies in those zones to increase NPP, and land use/cover change, such as afforestation, is an alternative policy that can be made.

## Conclusions

In this study, we selected the Three-North Shelterbelt Program (TNSP) zone in China, which accounts for more than 40% of China’s landmass, and has been the scene of several large-scale ecological restoration efforts since the late 1990s. MODIS annual Net primary production (NPP), MODIS land cover (MCD12Q1), and the Standardized Precipitation Evapotranspiration Index (SPEI) products for 2001–2010 were used to explain changes in NPP. We integrated correlation and decomposition analyse to determine the relative influence of drought and land cover conversion (LCC) on inter-annual changes of NPP. Our main findings are summarised as follows. (1) The 10-year average NPP in TNSP was about 420 Tg C. (2) We found that 60% of total annual NPP over the study area was significantly correlated (*p*<0.05) with SPEI, while the correlation was the highest for grassland.LCC had significant positive correlations (*p*<0.05) with NPP for most of land cover types, but not for grassland. (3) LCC, drought, and other natural or anthropogenic activities (ONA) contributed about 3%, 74%, and 23% of the total change in NPP, respectively. Rainfall is the most important factor for annual NPP variabilities in the TNSP zone, and the influence of LCC on NPP variability was limited, although many ecological restoration programs were carried out in this zone, because the area observed with LCC was much smaller than the whole TNSP zone. Our method quantitatively examined the relative roles of drought and LCC on changes in NPP. It is reasonable to improve the accuracy of the contributions of multiple mechanisms on NPP variability with more available higher resolution spatial remote sensing products, climate and anthropogenic data, and a combined method of scenario simulation and decomposition analyses.
